# Effect of Substituting Polyether Ionophore Anticoccidial Drugs With 1, 8‐Cineole for the Control of *Eimeria* Infections in Broilers

**DOI:** 10.1002/vms3.70341

**Published:** 2025-04-26

**Authors:** Jing Sun, Wanjun Bao, Ye Han, Rongfei Zhang, Zhijiang Zhou

**Affiliations:** ^1^ School of Chemical Engineering and Technology Tianjin University Tianjin The People's Republic of China; ^2^ Tianjin Naer Biotechnology Co., LTD. Tianjin The People's Republic of China

**Keywords:** 1,8‐cineole, anticoccidial activity, broilers, intestinal microbial flora

## Abstract

**Background:**

Coccidiosis is an important parasitic disease of broiler chickens. Drug resistance to the polyether polyether Ionophore anticoccidial drugs  (PACDs) caused by long‐term use has emerged as a significant problem in commercial broiler chicken production.

**Objectives:**

This study explored the feasibility of 1,8‐cineole (CIN) in replacing PACD with broiler feeding experiments and intestinal microecological experiments.

**Methods:**

This experiment selected 21‐day‐old Lingnan yellow (LNY) broilers and randomly divided them into 8 groups, with 30 chickens in each group. They were uniformly fed with full feed during the breeding period. The experimental groups were as follows: (i) three broiler groups that were fed a PACD supplementation (G1: Salinomycin Premix [SAP] 60 mg/kg; G2: Monensin Premix [MOP] 100 mg/kg and G3: Maduramicin Premix [MAP] 5 mg/kg), (ii) three broiler groups that were administered different CIN dosages (G4: CIN‐L 100 mg/kg, G5: CIN‐M 150 mg/kg and G6: CIN‐H 250 mg/kg) and (iii) two control broiler groups (G7: infected control group and G8: healthy control group). On the 23rd day, all groups were infected with coccidian cysts, except the G8 group which was used as a blank control. After 2 weeks of continuous feeding, the growth performance changes of broilers were analysed, and the intestinal lesions of eight groups of broilers were analysed after slaughter.

**Results:**

Compared with PACD treatment, the average daily feed intake (ADFI) of the three CIN‐treated groups increased by 13%, whereas the feed conversion ratio (FCR) reduced by 28%. FCR value for the high‐dose CIN treatment was 2.04, 34% decline which compared with control group infected with spore oocysts. The anticoccidial index (ACI) of all PACD treatments was less than 120, whereas the ACI of the middle‐ and high‐dose CIN treatments was higher than 180. In comparison to the *Eimeria*‐infected control group, the diversity and total number of the microbiota in the CIN treatments increased significantly. Moreover, CIN treatment favoured the proliferation of intestinal probiotics, especially *Lactobacillus* sp.

**Conclusions:**

CIN could have an inhibitory effect on parasite development as suggested in the gene function annotation of the intestinal microbiota. These results demonstrate that CIN may be a feasible natural alternative to anticoccidiosis drugs. So it was important to take precautions against and control coccidiosis in poultry production which has a lesser risk of creating resistance to substitutive PACDs.

## Graphical abstract

This study explored the feasibility of 1,8‐cineole extracted and separated from eucalyptus leaves in replacing PACD with broiler feeding experiments and intestinal microecological experiments. This experiment aims to investigate the effect of 1,8‐cineole in broiler chickens infected with coccidia through the evaluation of coccidia infestation and characterization of intestinal microbiota.

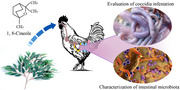

**Abbreviations**
ACI: anticoccidial index
ADFI: average daily feed intake
ADG: average daily gain
AGPs: antibiotic growth promoters
CIN: 1,8‐cineole
EO: essential oil
FCR: feed conversion ratio
LNY: Lingnan yellow
MAP: Maduramicin Premix
MOP: Monensin Premix
PACD: polyether Ionophore anticoccidial drug
RGR: relative weight gain rate
SAP: Salinomycin Premix
SCFA: short‐chain fatty acids
WGR: weight gain rate

## 1 Introduction

Coccidiosis is entirely common and serious parasitic disease in broilers. Poultry coccidiosis caused by *Eimeria* parasites results in considerable financial losses for the livestock industry (Taha et al. 2021). Coccidiosis can be caused by a variety of coccidia of the genus *Eimeria* parasitizing the small intestine or the cecal mucosa of broilers, where it multiplies and induces intestinal tissue damage and haemorrhage by being parasitic on cecal mucosal epithelial cells (Balacs 1997). *Eimeria tenella* causes cecal coccidia, has a strong pathogenic effect and mainly affects chicks between 3‐ and 5‐week‐old. Coccidia that damage the small intestinal mucosa (viz., the small intestinal coccidia) are also caused by *Eimeria*. In fact, there are seven species of coccidia that readily infect broilers. *Eimeria tenella*, *Eimeria necatrix*, *Eimeria brunetti*, *Eimeria maxima*, *Eimeria acerulina* and *Eimeria mitis* are the six main species that suffer significant economic losses from coccidiosis in animal husbandry (Williams 2002). The six main species behind significant financial losses (Lan et al. 2017). In recent years, there has been a notable increase in the frequency of clinical outbreaks of intestinal coccidiosis in broilers, resulting in significant losses to the poultry industry.

In order to improve the inhibition and restrain chicken coccidiosis, which seriously endangers the development of the chicken industry, it is necessary to select highly effective and sensitive anticoccidiosis drugs (Allen and Fetterer 2002). Most drugs have been used for some time, resulting in many different developments of resistance and susceptiveness loss (Kraieski et al. 2021). However, due to the widespread misuse of anticoccidial drugs in clinics, these drugs not only fail to effectively control coccidiosis but also increase feeding costs and contribute to the development of drug and cross‐resistance, thereby also resulting in a large number of drug‐resistant strains emerging (Chapman 1997).

Polyether anticoccidial drugs (PACDs) have been available for several years. Currently, salinomycin, monensin and maduramycin are widely used in the market. These drugs are often used as anticoccidial additives in broiler diets and inevitably interact with other drugs. At the same time, due to their narrow safety range, careless dosing could cause poisoning. As treatment time increases, PACDs lose their initial sensitivity. PACDs are similar in chemical structure and modus operandi; as a result, strains resistant to one of these drugs may also be resistant to other PACDs. However, among the currently used anticoccidial drugs, the antibiotic resistance rate of PACDs is slower than that of chlorphenidine, sulpha drugs, changshanone, aminoproline or nicarbazine; all of which are commonly used anticoccidial drugs (Idris et al. 2023).

Eucalyptus leaves contain a large number of active ingredients. Among them, 1,8‐cineole (CIN) is the basis ingredient (Wildy et al. 2000). CIN (C_10_H_18_O) has a molecular weight of 154.25, containing cyclic ether and alcohol functional groups, soluble in ether, chloroform, propylene glycol and other organic solvents and slightly soluble in water. Therefore, it has good fat solubility and penetrability to cell membrane (Merghni et al. 2023).

Recent studies have examined CIN for some biological and pharmacological properties, consisting of its disinsection characteristic (Thâmarah et al. 2021), mucolytic, anti‐microbial (Ralph et al. 2023), anti‐ageing, antineoplastic (Moteki et al. 2002), reducing inflammatory responses of gastric ulcer (Germana et al. 2015) and improving cardiac function (Wang et al. 2024).

In view of the nature and potential medical treatment prospects of CIN, we hypothesized that CIN has an effect on the control and prevention of coccidiosis in broilers. In this study, we infected young broilers with four common coccidiosis cysts and then fed different dosages of CIN supplemented feed for feeding to observe the control effect of CIN on coccidiosis in young broilers. The effect of CIN and PACDs on controlling coccidiosis in young broilers was compared. The mechanism of CIN controlling coccidiosis in young broilers was discussed. The study might provide the basis for exploring the use of plant extracts to control parasitic diseases in broiler chickens.

## 2 Materials and Methods

### 2.1 Strains and Materials

The strains used in this study were *E. tenella* ETWH, *E. necatrix* ENYS, *Eimeria acervulina* EAWY and *E. maxima* EMHL. All these strains were isolated and preserved in the Parasitic Biology Laboratory of Institute of Animal Science. The used PACDs (including Salinomycin Premix [SAP], Monensin Premix [MOP] and Maduramicin Premix [MAP]) were purchased from Company. Although 1‐day‐old Lingnan yellow (LNY) broilers were fed. The complete formula broiler feed (without any anticoccidial or antibacterial drugs) was specially produced by ourselves (Tables S1 and S2). All animal trials have been approved by the IACUC.

The experimental cage in this study is made of stainless steel with a size of 180 × 80 × 45 cm^3^. The feeder is made of polyethylene plastic and with a size of 530 × 350 mm^2^. The drinker is a nipple drinker, the length of the nipple is 20 mm, and the water pressure is 0.1–0.3 MPa.

### 2.2 Preparation and Feeding of CIN

CIN (content ≥90%) was purchased from France. Because CIN is insoluble in water, it is difficult to be absorbed by direct feeding of broilers. Before feeding, it is made into an emulsion with twice the volume of Tween 80. Specifically, Tween 80 is added to the right amount of distilled water and stirred at 500 rpm to disperse it evenly in the water, forming a clear or translucent solution. Then, the CIN drops were slowly added to the above Tween 80 solution, and 30 mn was stirred at 800 rpm during the drip process to make CIN fully and evenly mixed with Tween 80 solution to obtain a stable CIN emulsion. CIN emulsion was slowly added to the drinking water system through the dosing device, and the experimental broilers drank freely and monitored to make it reach the designed drinking amount.

### 2.3 Animal Experiment Design

Our experiment design was conducted refer to minor modifications (Mathis et al. 2021). The broilers were raised to the age of 21‐day (180–190 g) and were individually weighed. The broilers were divided randomly into 8 groups with 30 each group, and the experimental forms were as follows (Table S3): (i) three broiler groups that were fed with PACDs supplement (G1: SAP 60 mg/kg; G2: MOP 100 mg/kg and G3: MAP 5 mg/kg), (ii) three broiler groups that were administered different CIN dosages (G4: CIN‐L 100 mg/kg, G5: CIN‐M 150 mg/kg and G6: CIN‐H 250 mg/kg) and (iii) two control broiler groups (G7: infected group and G8: healthy group). All groups were oral administration of inoculation with 1.5 × 10^5^ sporulated oocysts, except the G8 group. Broilers were fed CIN and PACDs containing feed, infected with spore oocysts 2 days later and continued to be fed the above feed and observed for 12 days. The defecation and faecal status of broilers were observed daily. The weight of broilers were record. During this time, the experiment broilers were fed two to three times a day and fed freely. This study was carried out in July in Guangdong Province, China.

### 2.4 Broiler Growth Performance Test Indicators

Generally, the date of weight gain rate (WGR), average daily gain (ADG), average daily feed intake (ADFI), feed conversion ratio (FCR), relative weight gain rate (RGR) and survival rate (SR) were calculated as follows (Stephan et al. 1997):

(1) $$\mathrm{WGR}\;\left(\%\right)=\frac{\mathrm{final}\;\mathrm{weight}-\mathrm{initial}\;\mathrm{weight}}{\mathrm{initial}\;\mathrm{weight}}\times 100$$

(2) $$\mathrm{ADG}=\frac{\mathrm{final}\;\mathrm{weight}-\mathrm{initial}\;\mathrm{weight}}{\mathrm{test}\;\mathrm{days}}$$

(3) $$\mathrm{ADFI}=\frac{\mathrm{final}\;\mathrm{weight}-\mathrm{initial}\;\mathrm{weight}\;\mathrm{of}\;\mathrm{feed}}{\mathrm{test}\;\mathrm{days}}$$

(4) $$\mathrm{FCR}=\frac{\mathrm{feed}\;\mathrm{consumption}}{\mathrm{weight}\;\mathrm{gain}}$$

(5) $$\mathrm{RGRn}(\%)=\frac{\mathrm{WGR}\;\mathrm{of}\;\mathrm{infected}\;\mathrm{group}}{\mathrm{WGR}\;\mathrm{of}\;\mathrm{no}\;\mathrm{infected}\;\mathrm{group}}\times 100$$

(6) $$\mathrm{SR}\;(\%)=\frac{\mathrm{number}\;\mathrm{of}\;\mathrm{surviving}\;\mathrm{broilers}}{\mathrm{initial}\;\mathrm{number}\;\mathrm{of}\;\mathrm{broilers}}\;\times \;100$$

### 2.5 Evaluation of Anticoccidial Activity

As indicated by Suo's method (Suo et al. 1994), the blood stool scoring mode was applied at 120 h after the onset of the infection, and the proportion of blood in the stool was observed, photographed and recorded. The blood in the stool was scored 12–24 h after the infection with the *Eimeria* strains, according to the following criteria: ‘0’ means that the stool is blood‐free; ‘1’ means that 25% are bloody stools; analogously, ‘2’ means 50%, ‘3’ means 75% and ‘4’ means 100%.

On the 7th day after the infection, the broilers were slaughtered. According to the lesion scoring mode designed by Barwick et al. (1970), the intestinal lesions of each broiler were scored, and the lesion scores were converted into lesion values recorded as 0, 1, 2, 3 and 4. In the employed lesion score, when condition of the cecal lesions is different between two sides, the more seriously damaged side prevails.

The grading was as follows: ‘0’ indicates no obvious lesions and is healthy, ‘1’ indicates the existence of few scattered petechiae on the cecum wall, the intestinal wall not being thickened and the contents being normal; ‘2’ indicates the number of lesions being large, the contents of the cecum being obviously bloody, the wall of the cecum has a bit thickened, and the contents were unremarkable; ‘3’ indicates a lot of blood in the cecum or inner the a cecal core (blood condenses into pieces or off‐white, cheese‐like and banana‐like‐shaped mass), the cecal wall being hyper‐trophic and the amount of faeces in the cecum being low; ‘4’ indicates the cecum swelling due to a large amount of blood or intestinal core and the intestinal core containing or not containing faecal residue. Broilers that died due to coccidiosis were also calculated as ‘4’.

The lesion value was calculated according to the following equation:

(7) Lesionvalue=averagelesionscore×10

The faeces of 108 h after the infection were collected, stirred evenly and weighed. Three samples were taken from each group. The faecal oocysts were counted according to the McMaster's counting method, and the number of faecal oocysts per gram (OPG), as well as the oocyst value in each group, were calculated (Table S4) (McDougald et al. 1987).

### 2.6 Anticoccidial Index (ACI)

According to the calculation formula (Chapman 1998), the ACI was calculated as described in the following equation:

(8) $$\mathrm{ACI}=\left(\mathrm{RGR}+\mathrm{SR}\right)\times 100-\left(\mathrm{Lesion}\;\mathrm{value}+\mathrm{oocyst}\;\mathrm{value}\right)$$

he drug‐sensitive efficacy standard of ACI is that ACI < 160 is judged as positive (+), ACI ≥ 160 judged as negative (−) with low degree of drug resistance, 160 ≤ ACI < 180 indicated moderate drug resistance, ACI ≥ 180 indicated high drug resistance.

### 2.7 Detection of Microbial Levels in the Gastrointestinal Tract of Broilers

Different intestinal content was collected as samples to study the microbial flora of broilers. The contents of the intestines (duodenum and cecum) were collected from four broilers of each group randomly. All the samples were preserved in sterile tube and stored immediately at −80°C. Finally, 30 ng of the qualified genomic DNA samples were taken out. The V3–V4 area was amplified by PCR (forward primer: 5′‐ACTCCTACGGGAGGCAGCAG‐3′; reverse primer: 5′‐GGACTACHVGGGTWTCTAAT‐3′). To facilitate the detection of fragment range and library concentration, an Agilent 2100 bioanalyzer was used. Eligible gene libraries were conformed to a suitable platform, HiSeq/MiSeq (The Beijing Genomics Institute, Shenzhen, China).

### 2.8 Analysis of the Composition and Diversity of the Microbial Flora

Raw data were filtered to generate high quality clean reads, tags connected, OTU clustered, OTU taxonomy annotated and α‐ and β‐diversity analysed. The composition of species was visualized by the circlize package of R (VER NO.3.6.3). Shannon and Chao indices represent diversity and richness worked out by QIIME (VER NO.1.7.0). In order to work out the metaged UniFrac, the QIIME software (VER NO.1.9.1) was also used. Then most core coordinate analysis was confirmed so as to obtain the coordinates and to visualize for complicated, multi‐dimensional data packed through the WGCNA and the ggplot2 parcel in R (VER NO.2.15.3).

Functional genes were classified according to KEGG, and the predicted results of the KEGG function abundance of the bacterial community were obtained by using PICRUSt2 (VER NO.2.2.0.b) and R (VER NO.DABAO 3.4.10).

### 2.9 Statistical Analysis

All data have been expressed as mean ± standard error (SE). One‐way analysis of variance (ANOVA) was performed using Origin2018 (OriginLab, Northampton, USA) and IBM SPSS Statistics 26 (IBM Corp., Armonk, NY, USA), and the differences were considered termed significant (*p* < 0.05).

## 3 Results

### 3.1 Growth Performance of Broilers

The growth performance of broilers is shown in Table 1. During the experiment, the disposition, water consumption and faeces of the broilers of the healthy control group (G8) were normal. The feed intake in the G7 group was found to be significantly decreased (−27.40%; *p* < 0.05) after the infection with coccidias compared to the G8 group. The feed intake of the CIN‐treated groups was significantly improved, respectively, when stack up against the G7; *p* < 0.05), whereas only the PACDs have demonstrated significant improvements (12.74%) in that same respect. The weight gain of broilers has shown that the WGR values of CIN groups were significantly increased, but no obvious difference was discovered in G1, G2 and G3. After 14 days of feeding, the FCR of the CIN group's broilers was significantly reduced. In fact, the FCR of the G6 group (supplemented with 250 mg/kg CIN) was found to be reduced by 33.98% when compared with the G7 group. Although feed intake decreased in infected PACDs and CIN intervention groups, the feed intake of CIN treatment group recovered effectively. Furthermore, the feed conversion rate also be decreased. We can conclude that CIN may have a positive effect on the performance of coccidia‐infected broilers, especially restoring feed intake.

**TABLE 1:** Performance data of broilers fed different diets and treatments.

Group	Feed intake (kg)*	Average daily feed intake (kg)	Weight gain (kg)**	Weight gain rate (%)	Average daily gain (kg)	Feed conversion ratio	Relative weight gain rate (%)
G1	10.42 ± 0.47^ab^	0.74 ± 0.03^a^	3.73 ± 0.17^c^	0.66 ± 0.04^ab^	0.27 ± 0.02^b^	2.80 ± 0.13^d^	62.28
G2	11.15 ± 0.53^c^	0.80 ± 0.05^bc^	3.35 ± 0.13^ab^	0.60 ± 0.03^a^	0.24 ± 0.03^a^	3.23 ± 0.13^e^	55.87
G3	10.8 ± 0.37^ab^	0.77 ± 0.02^ab^	3.30 ± 0.09^a^	0.59 ± 0.03^a^	0.24 ± 0.06^a^	3.34 ± 0.11^e^	55.81
G4	13.04 ± 1.26^e^	0.93 ± 0.10^d^	10.82 ± 0.47^e^	0.92 ± 0.07^c^	0.37 ± 0.10^c^	2.52 ± 0.14^c^	86.58
G5	11.93 ± 0.73^cd^	0.85 ± 0.04^c^	10.99 ± 0.36^e^	0.99 ± 0.05^c^	0.39 ± 0.0^5^	2.18 ± 0.09^ab^	91.29
G6	11.46 ± 0.43^c^	0.82 ± 0.04^c^	11.2 ± 0.43^ef^	1.01 ± 0.12^c^	0.40 ± 0.03^c^	2.04 ± 0.07^a^	93.97
G7	9.89 ± 0.28^a^	0.71 ± 0.02^a^	8.87 ± 0.26^d^	0.57 ± 0.04^a^	0.23 ± 0.04^a^	3.09 ± 0.16^de^	53.55
G8	13.5 ± 0.94^e^	0.96 ± 0.13^d^	11.6 ± 0.64^ef^	1.07 ± 0.11^cd^	0.43 ± 0.05^cd^	2.26 ± 0.10^ab^	100

*Note*: Means within a row with different superscripts (a–f) are significantly different (*p* < 0.05).

* Total feed intake of 30 broilers for 14 days in each group.

** Total weight gain of 30 broilers for 14 days in each group.

During the 14 days of feeding, the broilers infected with spore oocysts gradually lost appetite and developed poor mental health. The amount of food and water intake was decreased to varying degrees in the infected groups. The feed and water intakes of the broilers belonging to the four infected groups were seriously reduced by the 5th and the 6th day. There were no dead broilers in any of the eight test groups.

### 3.2 Inhibitory Effect of CIN on Coccidia

The inhibitory effect of CIN on coccidia in broilers is shown in Table 2. The results showed that oocyst value in CIN group was significantly reduced, and oocyst value in medium‐ and high‐dose groups even dropped to 0, and the intervention effect was unexpectedly better than that in PACDs group.

**TABLE 2:** Results of growth performance and anticoccidial index (ACI) of broilers infected with mixed *Eimeria* strains.

Group	Initial quantity	Survival quantity	Survival rate (%)	Lesion value	Oocyst value	AC1 value	Drug resistance
G1	30	30	100	28	40	94.28	**+**
G2	30	30	100	36	40	79.87	**+**
G3	30	30	100	27	25.67	103.14	**+**
G4	30	30	100	25.6	11.67	149.31	**+**
G5	30	30	100	5.6	0	185.69	−
G6	30	30	100	3	0	190.97	−
G7	30	30	100	33	40	80.55	+
G8	30	30	100	0	0	200	−

*Note*: ACI < 160, it is judged as positive (+); ACI ≥ 160, it is judged as negative (−), indicates low drug resistant; 160 ≤ ACI < 180 indicates intermediate drug resistant; ACI ≥ 180 indicates high drug resistant.

### 3.3 Alleviating Effect of CIN on Cecum Lesion

**FIGURE 1:**
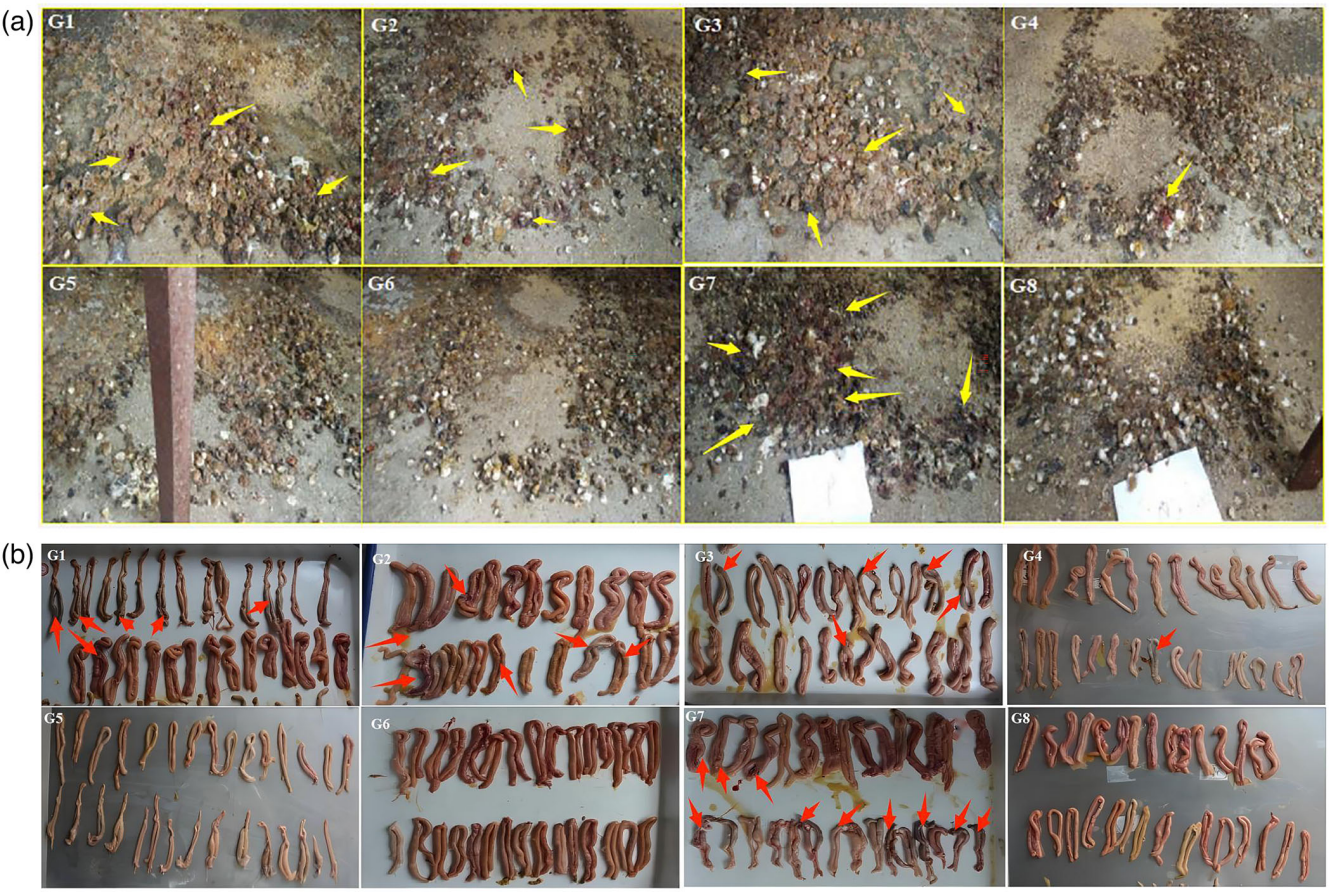
Blood stools in faeces of broilers infected with coccidian after 108 h (a) and cecum lesions of broilers infected with coccidian after 120 h (b). G1–G8 represent G1 group to G8 group, respectively. The yellow arrows point to the bloody stools, and red arrows point to the lesion.

After CIN feed intervention and coccidian cyst infection 12 days, all experimental broilers were dissected, and the damage to their small intestine and cecum was observed, mainly including the appearance of bloody stool and intestinal lesions. The bleeding stools of experimental broilers were observed 108 h after infection with coccidian cysts (Figure 1a). The area of blood in the G1, G2 and G7 groups exceeded 75%, whereas the area of blood in the G3 group was more than 50%, and the area of blood in the G4 group was less than 25%. No pathological changes were observed in the intestines of healthy non‐infected broilers G8 group. No blood was found in the G5, G6 and G8 groups. The lesion value of cecum was measured after broiler slaughtering (Figure 1b). The lesions infected with coccidia were eventually concentrated in duodenum and cecum. Slight pathological changes were observed in the low dosage G4 group with a small number of bleeding spots on the mucosal surface of the duodenum and hyperaemia and swelling of the intestinal wall. Although the pathological changes alleviate significantly in middle dosage G5 group and high dosage G6 group, the intervention effect of CIN was obviously superior to PACDs.

### 3.4 CIN Significantly Increased ACI

In order to better reflect the anticoccidia effect of CIN, a resistance characteristic ACI was determined and analysed. The results indicated that the ACI values of G1, G2 and G3 groups were all less than 120, exhibiting an ineffective anticoccidial effect. The ACI value of G4 group was 149.31, belonging low‐efficiency anticoccidial effect. The ACI values of G5 and G6 groups were higher than 180, undoubtedly being high anticoccidial effect, which approached the level of G8 group (Table 2). The result showed that the ACI of G1, G2, G3 Gand G4 groups was positive with drug resistance, whereas that of G5 and G6 groups was negative without drug resistance.

### 3.5 CIN Induced Changes of Intestinal Flora in Broilers

**FIGURE 2:**
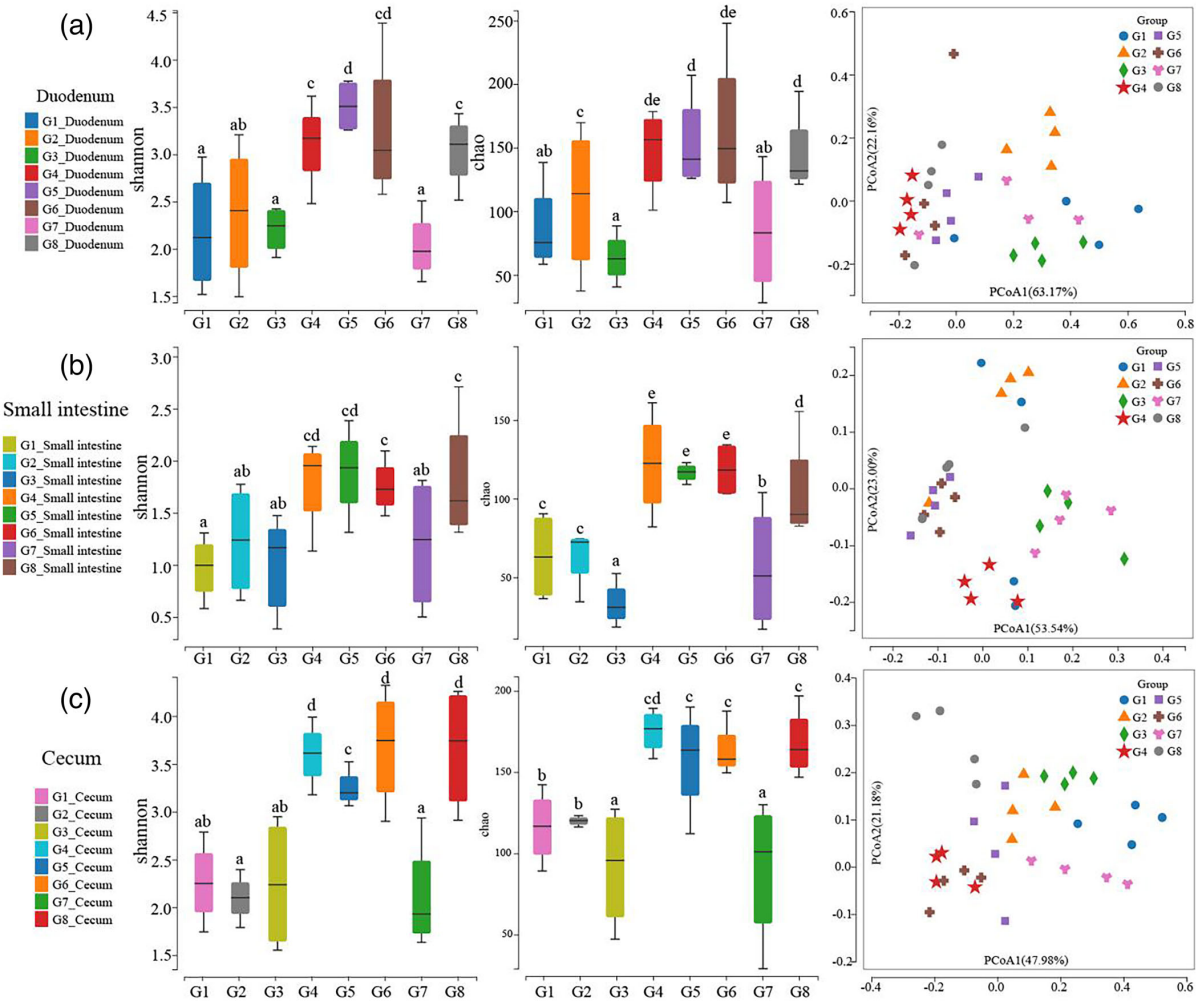
The Shannon value, Chao value and PCoA of microbial flora in duodenum (a), small intestine (b) and cecum (c) of broilers.

The bacterial flora in duodenum, small intestine and cecum contents of broilers treated with CIN were studied by 16s rDND metagenomics (Figure 2a–c). The main effects analysis indicated that G8 healthy control group and G7 test control group demonstrated a notable margin (*p* < 0.05) in terms of their Shannon value and Chao index. Compared to the G7 group, the Shannon value and the Chao index were also found to be significantly reduced (*p* < 0.05) in the PACD‐treated groups, meaning that the total number and the diversity of the intestinal flora decreased. Conversely, the Shannon value and the Chao index of the CIN‐treated groups were found to be significantly increased (*p* < 0.05), especially high dosage group.

**FIGURE 3:**
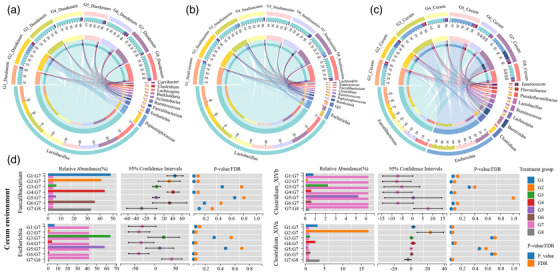
Dominant genera in the intestines of broilers in the duodenum (a), small intestine (b) and cecum (c and d) of broilers.

To further investigate the changes in the key microflora in each group, core microbiota analysis was applied to demonstrate the dominant microorganisms in the groups (Figure 3a–c). After broilers were infected with coccidia, the intestinal *Acinetobacter* and *Escherichia* increased significantly, whereas the healthy group, CIN intervention groups and PACDs intervention groups did not observe this phenomenon. The *Lactobacillus* was significantly stimulated (*p* < 0.05) in the CIN groups compared to infected group in small intestine of broilers. After CIN intervention, the abundance of *Faecalibaculum* (Figure 3c) significantly increased, with a more pronounced effect observed in the low/high‐dose groups in cecum of broilers. Compared with coccidian‐infected broilers in G7 group, the relative abundance of *Clostridium* X1Vb in all experimental groups was significantly decreased (*p* < 0.05), especially in G4, G6 and G8 groups.

### 3.6 Prediction of CIN Affecting the Function of Intestinal Flora

**FIGURE 4:**
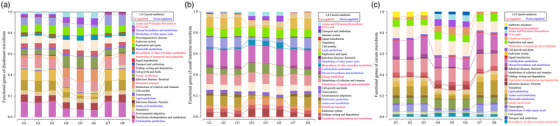
The predicted KEGG function in microbial flora of duodenum (a), small intestine (b) and cecum (c).

To elucidate the potential mechanism of CIN inhibition on coccidia growth, we performed KEGG prediction of intestinal function in infected broilers after CIN intervention (Figure 4).

The results have shown that the intestines of broilers, the Krebs cycle, membrane transport genes, metabolic genes and the supersession of terpenoids and polyketides, energy metabolism and membrane transport were all positively correlated with the CIN groups.

## 4 Discussion

### 4.1 Evaluation of CIN Inhibiting Coccidia Infection in Broilers

In this development, the appetite and feed intake of broilers that were infected with coccidia were seriously affected, and the intestinal absorption function of broilers with coccidiosis was severely impaired, resulting in an increase in feed conversion rate. At present, the main treatment method of broiler coccidiosis is to use the chemical drug PACDs, but the long‐term use of PACDs leads to the emergence of serious drug resistance. In recent years, many plant extracts have become potential candidates in search of alternative methods to treat coccidiosis in broilers. Some recent studies have shown that a CIN extracted from eucalyptus leaves has good fat solubility and therefore strong antibacterial and anti‐inflammatory effects (Aduayi et al. 2024). Therefore, we hypothesized that this fat‐soluble eucalyptus leaf extract may also have an inhibitory effect on coccidia.

In this study, we designed to treat and intervene young broilers infected with coccidia with different concentrations of CIN. During the trial, it was found that compared with healthy chickens, after infection, broilers had loss of appetite, decreased feed efficiency, significant weight loss and the feed intake of the G7 group infected with coccidia was 27.40% below than G8 group (*p* < 0.05). Furthermore, there were obvious bloody stool, a large number of coccidium oocysts in faeces and pathological changes of intestinal haemorrhagic spots and swelling.

The feed conversion rate increased, and the relative weight gain rate decreased when treated with PACDs. Although the intestinal damage value and oocyst number decreased in the high‐dose group, the treatment effect was not obvious in the medium–low‐dose group, and obvious drug resistance occurred in all the dose groups after PACDs treatment. After CIN intervention, the feed conversion rate of broilers decreased, the relative weight gain rate and body weight increased significantly, intestinal bleeding points of broilers were reduced, swelling was reduced, and no oocysts were found in faeces of the medium–high‐dose group, and drug resistance was not observed. The ADFI values of the three CIN groups significantly increased (*p *< 0.05), whereas their FCR values significantly decreased (*p* < 0.05). The FCR value of the high‐dose CIN‐treated group was 33.98% below than the G7 group.

ACI index is a comprehensive index to measure the ability of an anticoccidia drug to inhibit the growth and reproduction and reduce the harm of coccidia infection. After the use of a highly effective anticoccidia drug, it can significantly reduce the amount of oocysts in animal faeces and reduce the clinical symptoms caused, and correspondingly, its ACI index will be higher, indicating that the drug has strong anticoccidia efficacy. The ACI values of all PACD groups were less than 120, and the G2 group demonstrated the most obvious drug resistance in this study. The ACI values of the middle‐dose and the high‐dose CIN groups were higher than 180. It indicated that feed with CIN can be effectively protected against coccidiosis for broilers, but the addition amount should be equal to or exceed 150 mg/kg.

As for the inhibitory mechanism of CIN on coccidia, we speculated that it may be related to its damaging effect on cell membrane and anti‐inflammatory effect (Sun et al. 2018). Among them, CIN may interact with the membrane lipids of coccidia through its good lipid solubility, resulting in changes in membrane permeability and leakage of intracellular substances, thus killing the oocysts and inhibiting their reproduction (Domagoj et al. 2016). In addition, according to previous literatures (Martins et al. 2017), CIN's alleviating effect on intestinal mucosal inflammation may be through promoting the release of anti‐inflammatory factors, thus participating in the regulation of immune and inflammatory responses.

### 4.2 CIN Inhibited the Growth of Coccidia by Altering the Intestinal Flora

The gastrointestinal tract of domestic fowl is a sophisticated microecological system. These microbes affect considerably in the absorption of nutrients, the normal development of the intestinal morphology, the digestive function and the establishment of immune function and disease resistance (Leser and Mølbak 2009).

As for the inhibitory effect of CIN on coccidia, in addition to the direct inhibition on the growth of coccidia, it indirectly affected the growth of coccidia through the change of intestinal flora. In this study, it was found that CIN supplementation had different degrees of influence on microflora in small intestine and cecum of broilers. As a result of low pH and high flow rate of the intestinal environment, the abundance and diversity index of the small intestine and duodenum were low. Compared with G7 group, the Shannon value and the Chao index of the CIN‐treated groups were identified as significantly increased (*p* < 0.05); meanwhile, these same parameters were found to be significantly decreased in the PACDs (*p* < 0.05). The phenomenon showed that the diversity of the intestinal microflora of broilers was significantly decreased and that the total bacterial count was sharply decreased after the infection. Moreover, CIN could play a role in regulating the flora diversity and structure disrupted by coccidia. This study first demonstrated the role of CIN as an important factor in maintaining the stability of the broilers’ intestinal flora.

Our experiment showed that by adding CIN in the broiler feed, it can achieve an obvious anticoccidial effect. In addition, the CIN could maintain the stability of the intestinal flora of broilers infected with coccidian. However, coccidian was killed by PACDs, but some of the beneficial gut bacteria were also destroyed.

The digested matter then proceeds to the cecum, which is mainly to degrade the indigestible plant materials and to absorb water, glucose and volatile fatty acids. Therefore, the bacterial abundance and diversity index in the cecum were increased (Figure 3a–c). The species composition has shown that obligate anaerobes (including *Clostridium* XVIII, *Uminococcus* 2, *Lactobacillus*, *Bacillus*, *Lachnospira*, *Bifidobacterium* and *Subdoligranulum*) in the cecum were the most abundant. The relative abundance of *Faecalibacterium* in G7 group significantly reduced (*p* < 0.05) compared with G8 (Figure 3d), which were not significant in G5 and G6 groups. *Faecalibacterium* can bacterium ferment dietary fibre, promote the digestion and absorption of feed fibre components of broilers, produce short‐chain fatty acids (SCFAs), improve the physiological state of intestinal tract, promote the growth and development of intestinal villi and help broilers better absorb nutrients in feed and promote the growth of broilers. These results were consistent with our previously observed results. The relative abundance of *Escherichia* in G7 group significantly raise (*p* < 0.05) contrasit with G8 group, whereas there was no change (*p* > 0.05) in G3 and G5 groups.

In our study, the abundance of *Clostridium* X1Vb in the intestines of coccidian‐infected broilers after CIN treatment was significantly decreased compared to G8 group, as previous researchers found that *Clostridium* X1Vb may take advantage of the damaged intestinal environment to proliferate. The toxins produced by it will further aggravate the damage of intestinal mucosa, destroy the tight connection of intestinal epithelial cells and lead to increased intestinal permeability. In addition, *Clostridium* X1Vb has a synergistic effect with coccidia, causing more serious intestinal lesions in broilers, such as intestinal bleeding, ulcers and even necrosis. When the integrity of intestinal mucosa is damaged, the absorption of nutrients is seriously affected, and the growth performance of broilers will also decline.

We found that *Lactobacillus* was the dominant genus in the small intestine. Compared with G7, the abundance of *Lactobacillus* in the intestines of broilers after CIN intervention increased significantly. This may be due to the competitive adhesion of *Lactobacillus* to the mucosa surface of the intestinal tract and the production of organic acids and bacteriocins to inhibit the growth of harmful bacteria and maintain intestinal homeostasis. It can be used as a replacement to low level of antibiotics added in poultry feed (Ullah et al. 2021).

### 4.3 Effect of CIN on the Functional Annotation of Broiler Intestinal Microbiota

In our study, we have systematically investigated the impact of CIN on the function of the broiler intestinal microbes. This study found that CIN can significantly inhibit coccidia infection in broilers and inhibit coccidia growth and reproduction in broilers. The study found that in broilers infected with coccidia and fed CIN, it may be due to the fact that various nutrient substrates in the broiler intestinal tract can be more easily used by microorganisms. The comparatively abundance of related genes among the metabolization of terpenoids and polyketides in the intestinal tract of broilers was also found to be high; a fact that is related to the relative abundance of *Actinomycetes*, which was consistent with the above results. *Actinomycetes* are characterized by the decomposition of organic matter and the production of various natural drugs, enzymes and bioactive metabolites. In contrast to PACD, CIN is extracted from natural plant extracts and, therefore, can be easier to digest and metabolized. In general, supplementation of CIN to coccidian‐infected broilers could improve the health status of broilers by altering the intestinal flora and promoting the digestion and absorption of feed components.

## 5 Conclusions

Coccidiosis remains a huge threat to broiler farming, and search for more effective treatments to replace the chemotherapy drug PACDs remains one of the challenges facing the industry. This study showed that CIN, as a natural product, possessed good anticoccidial effect, no drug resistance, and could protect the stability of intestinal flora. CIN did not kill all bacteria like PACDS and can also improve the growth performance of broilers. Therefore, CIN as an alternative coccidiosis drug of eucalyptus leaf extract has a good application prospect in poultry breeding such as broilers. Therefore, it is recommended to feed CIN in the high temperature and high humidity season when coccidiosis is high, so as to prevent and control the occurrence of coccidiosis in poultry more effectively.

## Author Contributions

All authors contribute to this study.

## Ethics Statement

This study does not violate and does not involve moral and ethical statement. The animal experiments involved in this study have been certified by the IACUC (Number: PT‐2021012).

## Conflicts of Interest

The authors declare no conflicts of interest.

### Peer Review

The peer review history for this article is available at https://www.webofscience.com/api/gateway/wos/peer‐review/10.1002/vms3.70341.

## Supporting information



Supporting Information

## Data Availability

All data generated or analysed during this study are included in this published manuscript.
