# Supplementary material for: Effect of Substituting Polyether Ionophore Anticoccidial Drugs With 1, 8‐Cineole for the Control of Eimeria Infections in Broilers
Source: Vet Med Sci. 2025 Apr 26;11(3):e70341. doi: 10.1002/vms3.70341 (PMC12032530; doi:10.1002/vms3.70341)
Supplement: Supplementary file 1 — Supporting Information [file VMS3-11-e70341-s001.docx]

**Supplementary Information**

**Title:** Effect of Substituting Polyether Ionophore Anticoccidial Drugs with 1, 8-Cineole for the Control of *Eimeria* Infections in Broilers

**Running title:** 1, 8 - Cineole Substituting Anticoccidials in Broilers

**This file includes:**

**Table S1 to S4**

**Table S1 The diet composition of broiler feed**

| Composition | Content (%) |
| --- | --- |
| Corn | 52.85 |
| Soybean meal of 43% protein | 37.9 |
| Vegetable oil | 4.8 |
| Calcium bicarbonate | 1.74 |
| Limestone powder | 1.27^a^ |
| Sodium chloride | 0.2^a^ |
| Methionine | 0.24 |
| Premix composition | 1^b^ |

^a^ Feed grade standard.

^b^ 1-14 d, 1 kg premix composition: Vitamin A,15000 IU; Vitamin D, 35100 IU; Vitamin E, 19.2 IU; Vitamin K, 32.4 mg; Vitamin B_1_, 1.2 mg; Vitamin B_2_,10.2 mg; Vitamin B_6_, 2.4 mg; Vitamin B_12_, 0.012 mg; calcium pantothenate, 12 mg; Niacin, 39 mg; Folic acid, 1.2 mg; Biotin, 0.189 mg; Choline, 700 mg; Cu (CuSO_4_∙5H_2_O), 8 mg; Mn (MnSO_4_∙H_2_O), 100 mg; Fe(FeSO_4_∙7H_2_O), 80 mg; Zn (ZnSO_4_∙7H_2_O) 60 mg; I (KI), 0.35 mg; Se (Na_2_SeO_3_), 0.15 mg.

**Table S2 The chemical composition (calculated value) of the broiler feed**

| **Chemical composition** | **Content (%)** |
| --- | --- |
| Crude protein | 23.55 |
| Crude fat | 3.00 |
| Crude fiber | 4.15 |
| Calcium | 1.00 |
| Total Phosphorus | 0.65 |
| Available Phosphorus | 0.41 |
| Sodium | 0.20 |
| Lysine | 1.20 |
| Methionine | 0.38 |
| Methionine + Cystine | 0.88 |
| Metabolizable energy | 3258 kcal/kg |

**Table S3 Broilers grouped according to the addition of anticoccidial substances in feed and coccidia infection**

| Groups | Diets and anticoccidial substances | Infection Time after addition of anticoccidial substances | Infected  **oocyst** dose |
| --- | --- | --- | --- |
| G1 | Basal diet+60 mg/kg SAP | 2 days | 1.5×10^5^ |
| G2 | Basal diet+100 mg/kg MOP | 2 days | 1.5×10^5^ |
| G3 | Basal diet+5 mg/kg MAP | 2 days | 1.5×10^5^ |
| G4 | Basal diet+100 mg/kg CIN-L | 2 days | 1.5×10^5^ |
| G5 | Basal diet+150 mg/kg CIN-M | 2 days | 1.5×10^5^ |
| G6 | Basal diet+250 mg/kg CIN-H | 2 days | 1.5×10^5^ |
| G7 | Basal diet | No Treatment | 1.5×10^5^ |
| G8 | Basal diet | No Treatment | No infected |

**Table S4 Conversion relationship between OPG and oocyst value**

| OPG (1×10^6^) | 0-1 | 1-25 | 26-50 | 51-75 | 76-100 |
| --- | --- | --- | --- | --- | --- |
| Oocyst value | 0 | 5 | 10 | 20 | 40 |
